# *LMNA* mutation leads to cardiac sodium channel dysfunction in the Emery-Dreifuss muscular dystrophy patient

**DOI:** 10.3389/fcvm.2022.932956

**Published:** 2022-07-22

**Authors:** Kseniya Perepelina, Anastasia Zaytseva, Aleksandr Khudiakov, Irina Neganova, Elena Vasichkina, Anna Malashicheva, Anna Kostareva

**Affiliations:** ^1^World-Class Research Centre for Personalized Medicine, Almazov National Medical Research Centre, Saint-Petersburg, Russia; ^2^Department of Embryology, Faculty of Biology, St Petersburg State University, Saint-Petersburg, Russia; ^3^Laboratory of Biophysics of Synaptics Processes, Sechenov Institute of Evolutionary Physiology and Biochemistry RAS, Saint-Petersburg, Russia; ^4^Institute of Cytology, Russian Academy of Sciences, Saint-Petersburg, Russia; ^5^Department of Women's and Children's Health and Center for Molecular Medicine, Karolinska Institute, Stockholm, Sweden

**Keywords:** lamin A/C, *LMNA* mutation, cardiomyopathy, induced pluripotent stem cells, sodium ion channel, cell differentiation

## Abstract

Pathogenic variants in the *LMNA* gene are known to cause laminopathies, a broad range of disorders with different clinical phenotypes. *LMNA* genetic variants lead to tissue-specific pathologies affecting various tissues and organs. Common manifestations of laminopathies include cardiovascular system abnormalities, in particular, cardiomyopathies and conduction disorders. In the present study, we used induced pluripotent stem cells from a patient carrying *LMNA* p.R249Q genetic variant to create an *in vitro* cardiac model of laminopathy. Induced pluripotent stem cell-derived cardiomyocytes with *LMNA* p.R249Q genetic variant showed a decreased sodium current density and an impaired sodium current kinetics alongside with changes in transcription levels of cardiac-specific genes. Thus, we obtained compelling *in vitro* evidence of an association between *LMNA* p.R249Q genetic variant and cardiac-related abnormalities.

## Introduction

The nuclear lamins belong to the type V intermediate filament proteins. The type A lamins encoded by the *LMNA* gene encompass lamin A and C isoforms, resulting from the *LMNA* gene alternative splicing (1), as well as more rare lamin C2 (2) and lamin AD10 (3) isoforms. A-type lamins are predominantly expressed in differentiated cells, including cardiomyocytes (CMs) (4). Lamins A and C are central elements of the nuclear lamina and are responsible for the proper nucleus architecture. Besides the structural function, lamins play a role in the regulation of gene transcription by direct and indirect modulation of the chromatin organization, DNA replication, and intercellular signaling (5–8). More detailed information about the functions of A-type lamins can be found in our recent review (9).

Mutations in the *LMNA* gene cause severe genetic diseases called laminopathies. Laminopathies are characterized by a wide range of clinical manifestations, predominantly affecting tissues of mesenchymal origin (adipose, bone, and muscle tissues) (10, 11). According to ClinVar database, 777 missense genetic variants have been described in association with different phenotypes. Three of them are classified as benign or likely benign, 184 are pathogenic or likely pathogenic, 80 have a conflicting interpretation of pathogenicity, and 480 are the variants of uncertain significance. Clinical significances of the rest 30 missense variants are not provided. Cardiac structure and conduction abnormalities are common symptoms of some laminopathies, such as Emery-Dreifuss muscular dystrophy (EDMD), Limb-girdle muscular dystrophy 1B, and Hutchinson-Gilford Progeria Syndrome (12, 13). Despite the recent advances in iPSC-based laminopathies modeling and animal modeling, the molecular basis of these diseases and the origin of their high phenotypic variability remain unclear.

Most *LMNA* mutations affect the striated muscles and result in the development of muscular dystrophies. In 1955, EDMD was described for the first time as a disorder with cardiac and skeletal muscle involvement (14). The majority of EDMD cases are associated with genetic variants in *LMNA, EMD*, and *FHL1* genes. Therefore, EDMD is a rare genetic disease characterized by progressive muscle weakness and atrophy, early contractures, cardiac conduction disturbances, and cardiomyopathies, eventually causing lethal arrhythmias (15, 16). The presence and severity of the clinical phenotypes are subtype-dependent and individual and could also include supraventricular and ventricular arrhythmias (15).

Currently, the detailed mechanism of *LMNA* genetic variants impact on the cardiac system is poorly understood. There are several hypotheses to explain the effect of the *LMNA* mutations on cardiac function. The structural hypothesis, proposed by Nikolova et al., suggests that *LMNA* mutations result in morphological abnormalities of a nucleus structure (17). According to the gene expression hypothesis, the main effect of *LMNA* mutations is mediated by the alterations in the chromatin organization, especially by the impaired formation of lamina-associated domains (LADs) (18, 19). In the mechanotransduction model, lamins are considered as a part of LINC complex (Linker of Nucleoskeleton and Cytoskeleton), which acts as regulatory machinery in contractile tissue, on the one hand, protecting the cell against mechanical stress (20) and, on the other, transmitting the extracellular mechanical signals to the nucleus (21).

Transgenic mouse models were widely used to study the role of *LMNA* genetic variants in cardiac conduction. Heterozygous *lmna*^+/−^ mice developed cardiac conduction disease and ventricular dilatation and had a shorter lifespan (22). *Lmna* knockout and haploinsufficient mice demonstrated defective force transmission, increased levels of pro-adipogenic factors, and downregulation of Wnt signaling (23). Transgenic mice carrying *Lmna*-H222P and *Lmna*-N195K reconstituted the dilated cardiomyopathy phenotype (DCM) in mice (24, 25). Despite a large body of information obtained from murine models, these data could not be directly translated into humans due to the differences in cardiac physiology between species.

Up to date, the most relevant approaches for cardiovascular research are based on iPSC-derived cardiomyocytes (iPSC-CMs). These cells successfully recapitulated disease phenotypes specific to the patient's genetic background. Recently, iPSC-CMs models were used to study cardiomyopathy-associated changes in ion channel function (26–29). For example, sodium current reduction was found in various inherited cardiac diseases such as DCM and cardiac conduction disorder (30), Brugada syndrome (31, 32), and arrhythmogenic cardiomyopathy (33). Alterations in the biophysical properties of sodium channels were described in iPSC-CMs model of Myotonic dystrophy type 1 associated with the expansion of a CTG repeat in the 3′ untranslated region of a cAMP-dependent protein kinase (DMPK). The patient's iPSC-CMs showed the downregulation of adult SCN5A isoform and subsequent depolarizing shift of steady-state activation kinetics of the sodium current (34). Salvarani et al. (35) reported that the *LMNA* p.K219T genetic variant was associated with changes in electrophysiological properties of CMs, including altered action potential, reduced peak sodium current, and diminished conduction velocity.

In the present study, we analyzed patient-specific iPSC-CMs to estimate the impact of the *LMNA* p.R249Q (*LMNA* R249Q) genetic variant on the expression of cardiac-specific markers and sodium current parameters. Recently, we generated and characterized the iPSC line FAMRCi007-A from the patient carrying *LMNA* R249Q genetic variant. This patient was diagnosed with EDMD accompanied with an atrioventricular block and paroxysmal atrial fibrillation (36). Genetic variant R249Q has previously been identified as pathogenic and associated with cardiovascular dysfunction (37).

Here, we demonstrated that *LMNA* R249Q resulted in alterations of cardiac-specific gene expression and changes of sodium current parameters, including a decrease in sodium current density, delayed activation, and impaired inactivation. Overall, these results contribute to a better understanding of the phenotypic diversity of cardiomyopathies caused by the *LMNA* genetic variants.

## Materials and Methods

### Generation and cultivation of human IPSCs

iPSC line with *LMNA* R249Q (FAMRCi007-A) was generated from the patient's PBMCs using CytoTune-iPS 2.0 Sendai Reprogramming Kit (Invitrogen, USA) as described previously (36). Detailed information about FAMRCi007-A line biological characteristics could be found in hPSCreg database (https://hpscreg.eu/cell-line/FAMRCi007-A). Additionally, two control wild type lines were used: AD3 and WTSIi004-A.

AD3 line was generated from human neonatal fibroblasts (hNFs) using the lentiviral, nonintegrating CytoTune-iPS 2.0 Sendai Reprogramming kit (Invitrogen, USA) according to the manufacturer's instructions. hNFs were purchased from Lonza (Slough, UK) and were cultured as described (38). Generated AD3 iPSC line was characterized according to the protocol published before (39) and fulfilled all criteria of pluripotency (38, 40).

WTSIi004-A line was obtained from European Bank for Induced pluripotent Stem Cells (EBiSC) (https://www.sigmaaldrich.com/RU/en/product/sigma/66540074). The EBiSC Bank acknowledges Wellcome Trust Sanger Institute (WTSI) as the source of the hiPSC line WTSIi004-A (HPSI1113i-qolg_3), which was generated with support from EFPIA companies and the European Union (IMI-JU').

All iPSC lines were cultured on plates coated with Geltrex in Essential 8 medium (all from Thermo Fisher Scientific, USA). Cells were passaged upon reaching 70–80% confluence using ReLeSR (Stem Cell Technologies) as a detaching reagent. For the next 24 h after passaging, the culture media was supplemented with Rock kinase inhibitor Y-27632 (5 μM, Tocris). iPSCs were maintaned at 37°C with 5% CO_2_ in a humidified incubator.

### Differentiation of IPSCs toward cardiomyocytes (CMs)

iPSC differentiation in CMs was performed by modulating Wnt pathway, applying small molecules (41) with modifications. In brief, iPSCs were seeded in density 6 × 10^5^ cell/cm^2^ 2 days before induction of cardiogenic differentiation. When cells reached 90–100% confluency differentiation was induced toward cardiogenic direction by changing growth medium (Essential 8) to differentiation medium [RPMI/B27ins-: Glutamax supplemented RPMI1640, B27 supplement without insulin, 100 U/ml penicillin, 100 μg/ml streptomycin (Thermo Fisher Scientific, USA)] with 6 μM CHIR99021 (Selleckchem, UK) (day 0). After 48 h previous medium was switched to RPMI/B27ins- medium containing 5 μM IWR1 (Stem Cell Technologies, Canada) (day 2). For the next 48 h, cells were cultured in RPMI/B27ins- medium. From day 6 to days 21–23 cells differentiated in RPMI/B27 medium [Glutamax supplemented RPMI1640, B27 supplement, 100 U/ml penicillin, 100 μg/ml streptomycin (Thermo Fisher Scientific, USA)]. RPMI/B27 medium was changed every 2 days. For electrophysiological analysis, from day 12 to day 15, metabolic selection of CMs was conducted as described previously (42). After the selection, cells were cultured in RPMI/B27 medium until seeding on Geltrex-coated coverslips for electrophysiological analysis of Nav1.5. Tryple Select reagent (Thermo Fisher Scientific, USA) was used to dissociate iPSC-CMs to single cell suspension.

Quantitative gene expression analysis and immunofluorescence staining were carried out at 0, 2, 5, 7, 14, and 21 days of cardiogenic differentiation.

**Figure 1:**
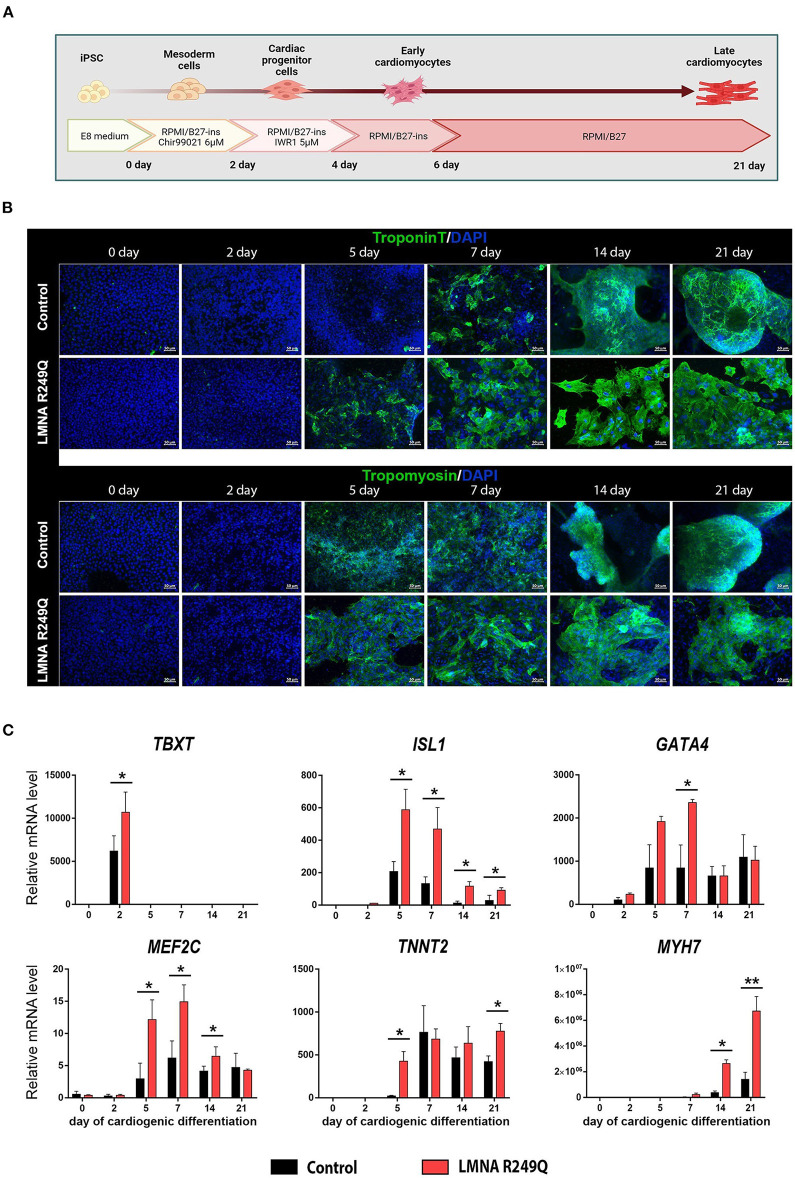
Cardiogenic differentiation of human induced pluripotent stem cells (iPSC) into cardiomyocytes. **(A)** Short scheme of cardiogenic differentiation consequent stages with descriptions of the medium content. **(B)** Immunofluorescence staining of iPSC (Control and *LMNA* R249Q) on a different stage of differentiation (0, 2, 5, 7, 14, 21 days) for cardiac-specific markers troponin T and tropomyosin. Scale bars, 50 μm. **(C)** Cardiac genes expression in Control iPSC-CMs and *LMNA* R249Q iPSC-CMs on different stages of cardiogenic differentiation as measured by qPCR. A value of 1 was given to mRNA levels for Control iPSC on day 0 of cardiogenic differentiation for all genes. The results are represented as mean ± SD; *p < 0.05, **p < 0.01.

A short scheme describing iPSC-CMs differentiation is presented in Figure 1A.

### Immunofluorescence staining

For immunofluorescence staining, cells were fixed with 4% paraformaldehyde for 12 min at room temperature, rinsed twice with phosphate-buffered saline (PBS). Using 0.5% Triton X-100 diluted in PBS, cells were permeabilized for 5 min. For blocking the non-specific binding, cells were incubated with 1% bovine serum albumin solution in PBS for 30 min, followed by washing with PBS. Then, the cells were incubated for 1 h with the primary antibodies: against human troponin T (Santa Cruz, USA) and human tropomyosin (Santa Cruz, USA). Secondary antibodies conjugated with Alexa488 (Invitrogen, USA) were used. For nuclei detection, cells were counterstained with DAPI (Sigma-Aldrich, USA) for 1 min. The coverslips with stained cells were mounted on glass slide with fluoromount (Sigma-Aldrich, USA). The staining was visualized using AxioObserver Z1 (Zeiss, Germany) microscope, and the image was processed using Zen Blue software (Zeiss, Germany).

### Gene expression analysis

Gene expression studies were performed on iPSC-CMs on 0, 2, 5, 7, 14, and 21 days of differentiation using qPCR. Total RNA was isolated from cells with Extract RNA reagent (Evrogen, Russia) and was subsequently treated with DNase I (Thermo Fisher Scientific, USA). Reverse transcription reaction was performed using MMLV RT kit (Evrogen, Russia). QPCR measurement was conducted using the qPCR mix-HS SYBR+LowROX (Evrogen, Russia) with gene-specific primers (Supplementary File) on an LC480 platform (Roche, Basel, Switzerland). The relative expression of target genes was calculated by the 2^−ΔΔCt^ method with the *GADPH* used as the reference gene.

### Sodium current recording

Sodium current traces were obtained using whole-cell patch-clamp configuration. For electrophysiological recordings, single-cell CMs seeded on Geltrex-covered coverslips were used. The extracellular solution for sodium current recording contained 140 mM NaCl, 1 mM MgCl2, 1.8 mM CaCl2, 10 mM HEPES, 10 mM glucose, pH 7.4 (CsOH). Nifedipine 10 mkM was added to the extracellular solution to exclude calcium currents. The intracellular solution contained 130 mM CsCl, 10 mM NaCl, 10 mM, EGTA, 10 mM HEPES, pH 7.3 (CsOH). Microelectrodes were manufactured using a puller (P-1000, Sutter Instrument). The electrode resistance ranged from 1.8 to 3.2 MΩ. Data acquisition and junction potential correction were done using Axopatch 200B amplifier and Clampfit software version 10.3 (Molecular Devices Corporation). Currents were acquired at 20–50 kHz and low-pass filtered at 5 kHz using an analog-to-digital interface (Digidata 1440A acquisition system, Molecular Devices Corporation). The series resistance was compensated at 75–80%. To avoid the possible time-dependent shift of activation and steady state inactivation curves, all pulse protocols were applied more than 5 min after membrane rupture. Current densities at each test potential were obtained by dividing the I_Na_ by cell capacitance. Cell capacitance values did not differ significantly between experimental groups. All measurements were performed at room temperature.

### Patch-clamp data analyses

Pulse protocols were applied with a holding potential of −100 mV. Current-voltage (I-V) curves were assessed by depolarizing voltage steps from −80 to 60 mV during 40 ms in 5 mV increments at 1 Hz frequency. The maximal *I*_Na_ at each voltage was obtained and the corresponding conductance (*G*) was calculated using equation *G* = *I*_Na_/(*V* – *V*_rev_), where *V* is the voltage test. The normalized *G* values were plotted against the voltage, and the *G*–*V* curves, which characterize the steady-state activation, were fitted to the Boltzmann function *G*/*G*_max_ = 1/(1 + exp [(*V*1/2 – *V*)/*k*)], where *G*_max_ is the maximal sodium conductance, *V*1/2 is the potential of half-maximal activation, and *k* is the slope factor. The voltage dependence of the steady-state inactivation was tested by measuring *I*_Na_ elicited by a 20 ms step to −15 mV after a prepulse of 500 ms ranging from −120 to 0 mV in 5 mV steps. The normalized *I*_Na_ was plotted against the prepulse voltage. The steady-state inactivation curves were fitted with the Boltzmann function.

## Results

### Generation of IPSC-based model of healthy CMs and *LMNA* R249Q CMs

iPSC-CMs carrying *LMNA* R249Q and control iPSC-CMs were generated according to the Wnt/β-catenin signaling pathway modulation protocol (Figure 1A). The duration of cardiogenic differentiation was 21–23 days for electrophysiological analysis. However, for immunofluorescence and qPCR analysis, the duration of differentiation varied (0, 2, 5, 7, 14, 21 days) to observe the expression of cardiac-specific markers in dynamic.

In the progress of differentiation, the iPSC-derived cardiomyocytes demonstrated gradually increasing levels of troponin T and tropomyosin, as was shown by immunostaining. We noted earlier expression increase of troponin T protein in iPSC-CMs with *LMNA* R249Q compared to control (Figure 1B). Any other changes related to structural abnormalities of iPSC-CMs carrying *LMNA* R249Q have not been observed.

To investigate whether the expression of cardiac-specific genes is altered in *LMNA* R249Q cardiomyocytes, we measured expression levels of the cardiac transcription factors *TBXT, ISL1, GATA4, MEF2C* and sarcomeric genes *TNNT2* and *MYH7* in control and *LMNA* R249Q iPSC-CMs by qPCR. Measurements were performed on different developmental stages of cardiogenic differentiation (0, 2, 5, 7, 14, 21 days) (Figure 1C). CHIR99021 treatment at the early stages of differentiation protocol activates mesoderm formation and thus markedly increases the expression of mesodermal marker *TBXT* (day 2). *LMNA* R249Q iPSC-CMs demonstrated significantly higher *TBXT* level compared to control iPSC-CMs. Expression of the essential early-stage cardiac genes (*ISL1, GATA4, MEF2C*) was detected after two days of differentiation. The *LMNA* R249Q was linked with elevated expression of these genes. In detail, increased *ISL1* level was observed in the *LMNA* R249Q iPSC-CMs on 5, 7, 14, and 21 days of differentiation. The *GATA4* level was increased in *LMNA* R249Q iPSC-CMs on day 7, and the *MEF2C* level was increased in *LMNA* R249Q iPSC-CMs on 5, 7, and 14 days of cardiogenic differentiation.

Expression of late-stage cardiac-specific markers (*TNNT2* and *MYH7*) was detected starting from 5 to 7 days of differentiation. *TNNT2* had a higher level in *LMNA* R249Q iPSC-CMs compared to control iPSC-CMs on day 5 and day 21 of differentiation. *MYH7* expression was significantly increased in *LMNA* R249Q iPSC-CMs compared to control iPSC-CMs on day 14 and day 21 of differentiation.

Taking together, *LMNA* R249Q iPSC-CMs demonstrated increased expression levels of mesodermal and cardiac-specific transcription factors as well as sarcomeric genes, indicating overall dysregulation of gene expression profile in iPSC-CMs carrying *LMNA* R249Q genetic variant.

**Figure 2:**
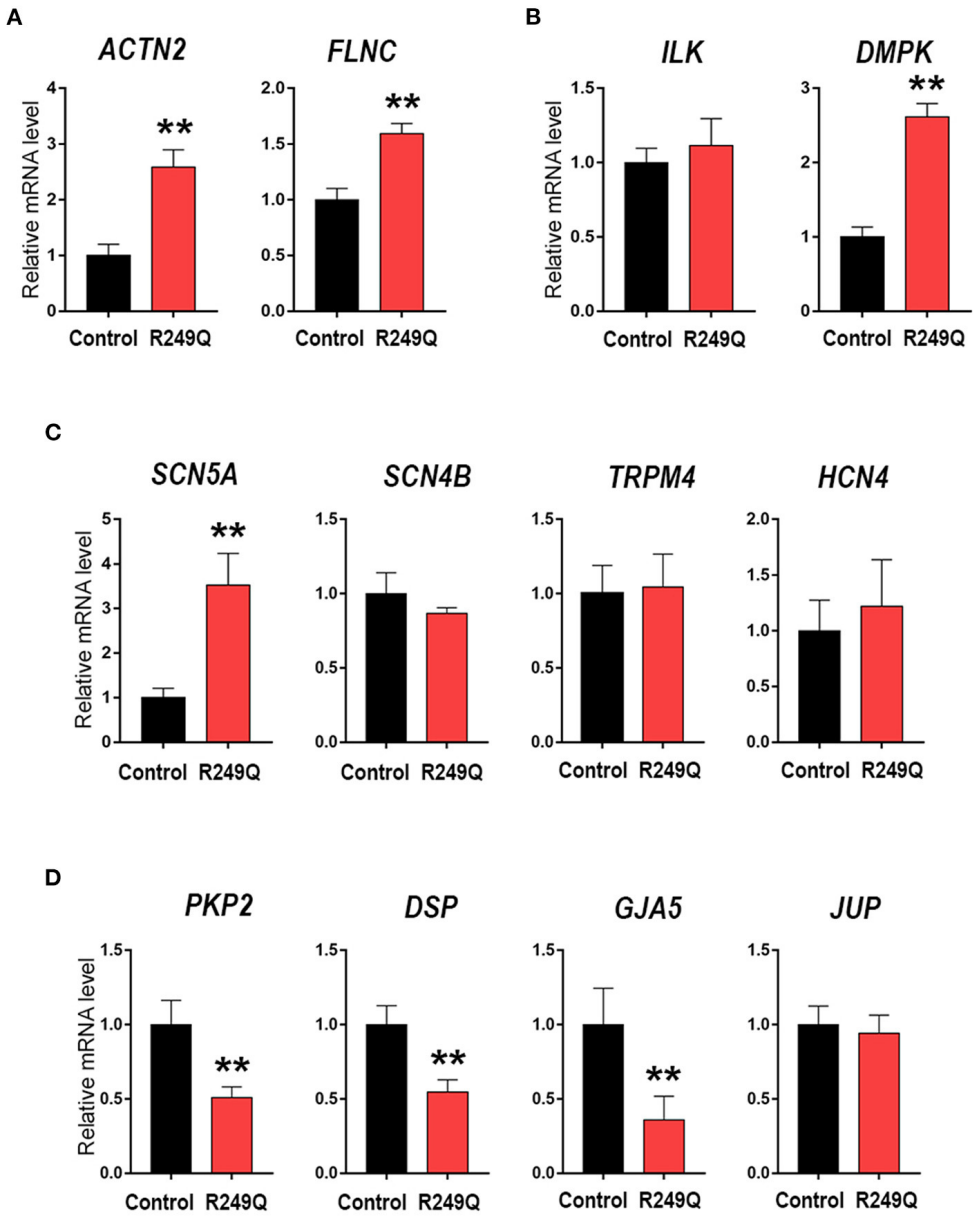
Cardiac genes expression in Control iPSC-CMs and *LMNA* R249Q iPSC-CMs on day 21 of the cardiogenic differentiation. mRNA level was measured by qPCR. The results are represented as mean ± SD; ** *p* < 0.01. **(A)** Level expression of the genes connected to sarcomeric cytoskeleton. **(B)** Genes encoding regulatory cardiac kinase. **(C)** Genes encoding ion channels. **(D)** Genes encoding proteins of an intercalated discs.

To elucidate whether the *LMNA* R249Q can affect gene expression in mature CMs we focused on the late-stage of cardiogenic differentiation (day 21) (Figure 2). We explored the expression of genes coding for sarcomeric proteins—*ACTN2* and *FLNC* (Figure 2A), and found a significant upregulation of these genes in CMs carrying *LMNA* R249Q compared to control CMs. In addition, we observed a significant increase in the expression level of the *DMPK* gene, encoding myotonic dystrophy protein kinase (Figure 2B), and increased expression level of the *SCN5A* gene, encoding cardiac voltage-gated sodium channel Na_v_1.5. However, any changes in the expression of genes coding for ion channels *SCN4B, TRPM4*, and *HCN4* have not been found (Figure 2C). Genes encoding components of intercellular contacts such as *PKP2, DSP*, and *GJA5* were downregulated in *LMNA* R249Q CMs (Figure 2D). Thus, observed complex alterations in the expression level of the key structural genes could contribute to the cardiac manifestation of the laminopathies.

### Functional characterization of voltage-gated sodium channel in the patient's cells

**Figure 3:**
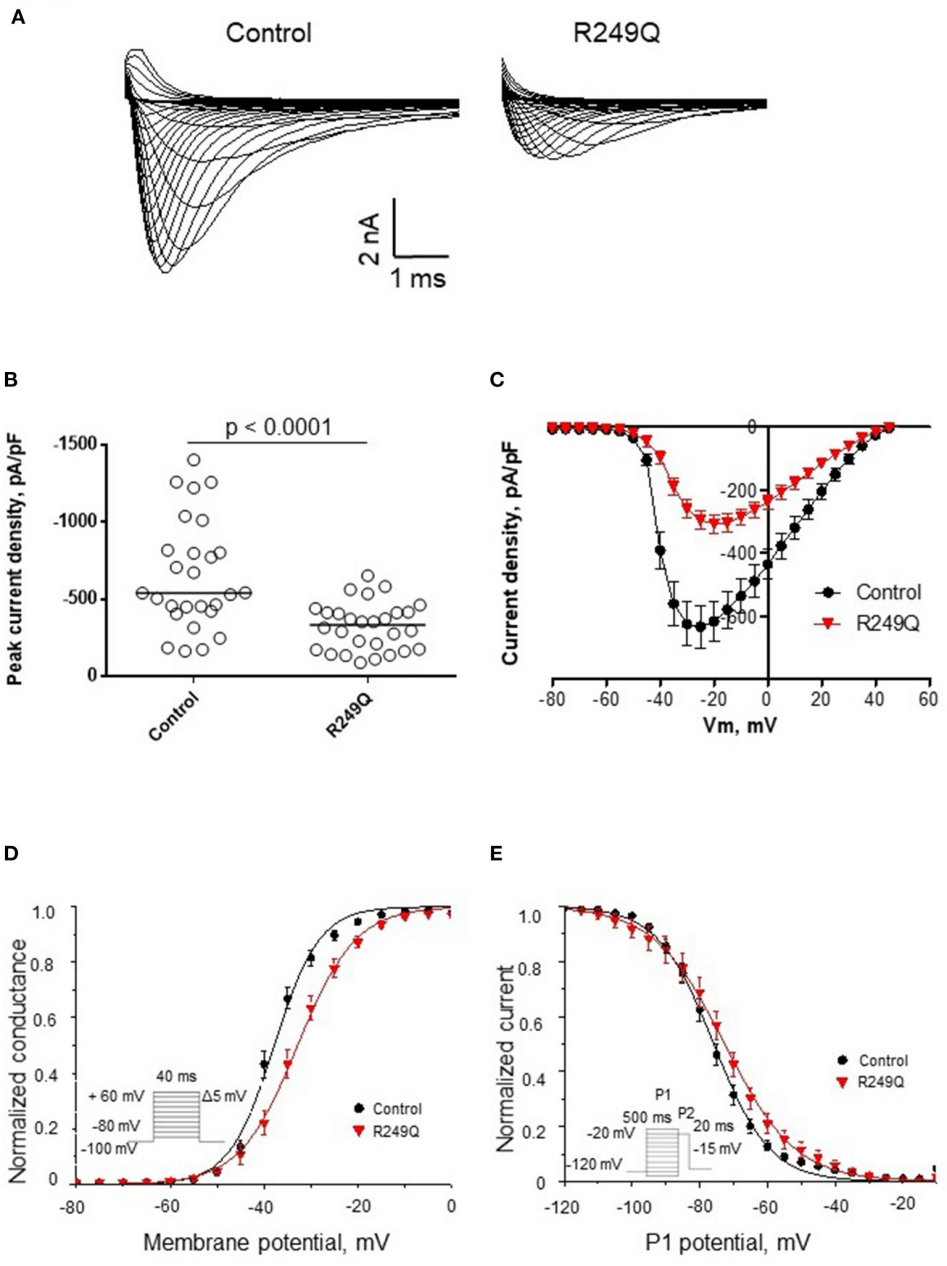
Functional characterization of sodium channel activity in iPSC-CMs carrying R249Q genetic variant in *LMNA* gene. **(A)** Typical sodium current traces in iPSC-CMs. **(B)** Peak sodium current densities for control and patient's iPSC-CMs. Data presented as medians. Patient's iPSC-CMs demonstrated marked decrease in sodium current density. **(C)** Current-voltage relationship of sodium channel in control and patient's iPSC-CMs. **(D)** Voltage-dependence of steady-state activation for control and patient's iPSC-CMs. Patient's iPSC-CMs showed delayed activation. **(E)** Voltage-dependence of steady-state inactivation for control and patient's iPSC-CMs. Patient's iPSC-CMs demonstrated impaired inactivation.

To define the functional effect of the *LMNA* genetic variant on the electrophysiological properties of iPSC-CMs, we performed sodium current recording *via* a patch-clamp technique. All experimental groups demonstrated typical sodium current traces (Figure 3A). The peak current density of iPSC-CMs from two different healthy controls did not differ significantly (Figure 3B; Table 1). However, patient's iPSC-CMs showed a marked reduction of peak current density compared with both controls. The analysis of the current-voltage relationship revealed that iPSC-CMs from both controls had their maximal current at −25 mV, whereas the patient's iPSC-CMs had a maximal current at a more depolarized potential of −20 mV (Figure 3C). It is well known that decreased current density may be caused by alterations in channel gating as well as protein trafficking disturbances.

**Table 1:** Biophysical parameters of sodium current in iPSC-CMs obtained from control and the patient's iPSC-CMs.

		**Control**	***n***	**R249Q**	***n***	***p***
Current density at −20 mV	pA/pF	−652.9 ± 68.4	27	−324.9 ± 29.3	28	*p* < 0.0001
Steady-state activation	*V*_1/2_, mV	−37.7 ± 0.8	27	−33.0 ± 1.2	28	0.002
	*k*	4.0 ± 0.3		4.7 ± 0.2		0.02
Steady-state inactivation	*V*_1/2_, mV	−75.7 ± 1.3	23	−73.4 ± 2.5	22	0.09
	*k*	7.1 ± 0.3		8.0 ± 0.4		0.07

*Capacitance. Control iPSC-CMs: 9.5 ± 0.85 pF; patient's iPSC-CMs: 11.4 ± 0.8 pF. p = 0.07*.

To examine the reasons for sodium current density reduction, we explored the kinetic parameters of the channel steady-state activation and inactivation. *LMNA* R249Q iPSC-CMs demonstrated a significant depolarizing shift of the voltage-dependence of steady-state activation compared to control iPSC-CMs (Figure 3D; Table 1). These changes in channel gating properties indicate loss-of-function of the sodium channel in patient-derived iPSC-CMs. Surprisingly, the voltage-dependence of steady-state inactivation was shifted to depolarized potential (Figure 3E; Table 1), which is typical for gain-of-function phenotype.

Thus, *LMNA* R249Q expression resulted in altered sodium channel functional activity of iPSC-CMs, reflecting the complex influence of the *LMNA* variant on the electrophysiological properties of the patient's cardiac myocytes. Therefore, observed changes in sodium current characteristics can be considered as possible mechanism of arrhythmia development in this patient.

## Discussion

Mutations in the *LMNA* gene, encoding A-type lamins, are known to cause heredity diseases with a wide range of phenotypes—laminopathies. One of the most frequent manifestations of these pathologies are heart rhythm and conduction abnormalities, including atrial fibrillation, ventricular arrhythmia, as well as sudden cardiac death. Dysfunction of a cardiac system can occur as a separate disease or as an accompanying pathology (22, 43–46). According to the gene transcription hypothesis (21), lamin A/C is considered a key regulator of gene expression. Therefore, we analyzed the expression profile of cardiac-specific genes in *LMNA* R249Q iPSC-CMs on the late-stage of cardiac differentiation. Obtained results showed the upregulation of the genes encoding the cytoskeletal proteins actin and filamin C in *LMNA* R249Q iPSC-CMs. The significant role of actin and filamin C in cardiomyocytes' physiology has previously been established (47). Both filamin C and actin were reported to contribute to the cortical cytoskeleton, thereby protecting the cardiomyocyte's structure from mechanical stress (48). Previously, DCM pathology was attributed to the disorganization of the intercalated discs and changes of cardiomyocyte shape. In particular, these changes refer to an increased expression of actin anchoring proteins suggesting an increased presence of filamentous actin (49). Considering that lamin A/C cooperates with actin *via* the LINC complex and participates in the regulation of the structural architecture of the contractile tissue (20), amino acid substitution in lamin A/C can impact sarcomere organization. In addition, lamin A/C, being involved in the regulation of the gene expression, can alter the proper transcriptional status of the cell because of *LMNA* genetic variants.

Furthermore, we demonstrated variation in cardiac genes expression in *LMNA* R249Q iPSC-CMs on the different stages of the cardiogenic differentiation. Since mutations in the *LMNA* gene lead to rearrangement of the spatial anchoring of genomic regions (referred to as LADs) to the nuclear lamina, its conformational changes are known to cause dysregulation of the gene expression (50). As a result, the earlier launch of the differentiation program can happen. In the present study, we found an enhanced cardiogenic differentiation capacity of the iPSC carrying the *LMNA* R249Q genetic variant. In the earlier report, an activation effect of the tissue-specific *LMNA* genetic variants on the adipogenic and osteogenic potential of human multipotent mesenchymal stromal cells was described (51). Another research established that both *LMNA* G232E/R482L variants caused dysregulation of muscle differentiation (52). From this perspective, we can assume that lamin A/C plays an essential role in the cell fate determination, and *LMNA* R249Q genetic variant leads to dysregulation of the differentiation program in the iPSC-CMs model.

Though clinical manifestations of cardiac arrhythmias and conduction abnormalities linked to *LMNA* variants have frequently been reported, there are only few reports of cardiac ion channels changes associated with *LMNA* genetic variants. In the present study, we determined that in *LMNA* R249Q iPSC-CMs, electrophysiological functions of cardiac sodium channel were markedly altered.

Recent work on iPSC-CMs revealed the effect of the *LMNA* K219T on the Na_v_1.5 function. *LMNA* K219T expression resulted in a decreased sodium current density and slower conduction velocity combined with reduced *SCN5A*, thus indicating an impaired activity of Na_v_1.5 channels (35). Our study found a similar effect of the *LMNA* R249Q variant on the Na_v_1.5 parameters, decreased sodium current density, and changes in sodium current kinetics. The genotype-phenotype correlation between *LMNA* genetic variant and resultant phenotype implies that pathologies caused by closely localized substitutions (both are localized in the rod domain of lamin A/C filament) share a common disease mechanism (53). However, in the present study, the expression of *SCN5A* in *LMNA* R249Q iPSC-CMs was increased, which could be a compensatory alteration. Probably even closely localized mutations in the *LMNA* do not share the completely identical pathogenic mechanisms *via* the epigenetically mediated regulation of *SCN5A* expression.

The question remains open whether the sodium current dysfunction is a primary or secondary consequence of the observed genetic variant in the *LMNA* gene. According to the expression data, *LMNA* R249Q iPSC-CMs had decreased levels of *PKP2, DSP*, and *GJA5*. The role of cell junction proteins in proper channel localization and function in intercalated discs is well established. Plakophilin and desmoplakin were reported to be integrated in the macromolecular complex of Na_v_1.5 in an intercalated discs pool of sodium channels (54). Na_v_1.5 was shown to be co-localized with plakophilin in intercalated discs, and the loss of *PKP2* expression resulted in sodium current reduction in culture of cardiac myocytes (55). The iPSC-CMs based studies of the effect of *PKP2* mutations on sodium current confirmed that PKP2 haploinsufficiency could lead to decreased sodium channel activity (33). Thus, in the current study, we further support the hypothesis that impaired expression of *PKP2* can lead to Na_v_1.5 dysfunction.

Along with the previous electrophysiological characterization of the *DSP* H1684R variant in iPSC-CMs linked to the decreased sodium and calcium currents (56), the present results underscore the importance of intercellular contact proteins in proper channel functioning. The role of the impaired expression of *GJA5* in sodium channel dysfunction remains unclear, despite the reported association of *GJA5* mutations with atrial fibrillation (57). Taken together, the sodium current reduction can be considered a consequence of *PKP2* and *DSP* deficiency in the *LMNA* patient cells. Precise molecular mechanisms underlying the electrophysiological defects in *LMNA*-associated cardiomyopathy remain unclear and demand further investigations.

To sum up, our results support the fundamental role of lamin A/C in cardiogenic differentiation, regulation of cardiogenic gene expression, and electrophysiology properties of the sodium ion channel.

## Data Availability Statement

The original contributions presented in the study are included in the article/Supplementary Materials, further inquiries can be directed to the corresponding author/s.

## Ethics Statement

The studies involving human participants were reviewed and approved by Almazov National Medical Research Centre (Approval No. 13/19.06.2014). The patients/participants provided their written informed consent to participate in this study. Written informed consent was obtained from the individual(s) for the publication of any potentially identifiable images or data included in this article.

## Author contributions

KP and AZ performed the experiments, drafted, and contributed to the writing of the manuscript. AKh provided helpful advice and critical reading of the paper. IN participated in the generation and characterization of the iPSC lines. AKo, AM, and EV contributed to the conception or design of the work, final approval of the version to be published. AKo and EV provided funding acquisition. All authors approved the final version of the manuscript.

## Funding

This work was financially supported by the Ministry of Science and Higher Education of the Russian Federation (Agreement No. 075-15-2022-301).

## Conflict of interest

The authors declare that the research was conducted in the absence of any commercial or financial relationships that could be construed as a potential conflict of interest.

## Publisher's note

All claims expressed in this article are solely those of the authors and do not necessarily represent those of their affiliated organizations, or those of the publisher, the editors and the reviewers. Any product that may be evaluated in this article, or claim that may be made by its manufacturer, is not guaranteed or endorsed by the publisher.

## Abbreviations

CMs: cardiomyocites
DCM: dilated cardiomyopathy
EDMD: Emery-Dreifuss muscular dystrophy
iPSC: induced pluripotent stem cell
LADs: lamina-associated domains
LINC: linker of nucleoskeleton and cytoskeleton
PBS: phosphate-buffered saline
PCR: polymerase chain reaction.
