# Supplementary material for: LMNA mutation leads to cardiac sodium channel dysfunction in the Emery-Dreifuss muscular dystrophy patient
Source: Front Cardiovasc Med. 2022 Jul 22;9:932956. doi: 10.3389/fcvm.2022.932956 (PMC9355377; doi:10.3389/fcvm.2022.932956)
Supplement: Supplementary file 1 [file Table_1.docx]

Supplementary Material

| **Gene** | **Forward/Reverse primer (5′-3′)** |
| --- | --- |
| *GAPDH* | AATGAAGGGGTCATTGATGG/AAGGTGAAGGTCGGAGTCAA |
| *TBXT* | GGGTACTCCCAATCCTATTCTGAC/ GCTGGACCAATTGTCATGGG |
| *ISL1* | GCGGAGTGTAATCAGTATTTGGA/GCATTTGATCCCGTACAACCT |
| *GATA4* | CGACACCCCAATCTCGATATG/GTTGCACAGATAGTGACCCGT |
| *MEF2C* | CCAACTTCGAGATGCCAGTCT/GTCGATGTGTTACACCAGGAG |
| *TNNT2* | TTCGACCTGCAGGAGAAGTT/GCGGGTCTTGGAGACTTTCT |
| *MYH7* | TAGACACACTTGAGTAGCCCAG/CTAGCCGCTCCTTCTCTGACT |
| *ACTN2* | GAACAAGATGGAGGAGATTGCC/GATGTTGTGCTCATACTGCTTCA |
| *FLNC* | GACAACCATGACTACTCCTACACT/CCGCCATAAGTCACTGTCACT |
| *DMPK* | GAGACCTATGGCAAGATCGTCC/CTGAATGAAGTCTCGAGCCTCC |
| *ILK1* | CCAAGATCAAGTGGCAGAGGAC/GTCCACAGGCATCTCTCCATAC |
| *SCN5A* | GAGCGGCTGTGAAGATTCTGGTTC/GCCATGAACACGCAGTTGGTG |
| *SCN4B* | GGCTCCTCCTTCTGTCTTAAGAG/CTGAGAGAAGCTGCATGATCCA |
| *TRPM4* | CTGCACGACGTTCATAGTTGAC/TGTGTGCATCGCTGTCCC |
| *HCN4* | CGCTGCCACCACTTCTGT/TTCAGGTGCCTTGGCGTC |
| *PKP2* | GCTGCTTCCGTCCTTCTGTA/GGAGTGGTAGGCTTTGGCA |
| *DSP* | GGCACCAGCAGGATGTACTA/CTCCTGGATGGTGTTCTGGT |
| *GJA5* | GCCAGTACTTCATCTACGGAATCT/GGATACGTAACAGTTGACCGG |
| *JUP* | ACTCTGTGCGTCTCAACTATGG/AAGCCGATGGTTGCCTTGAC |

**Supplementary Table.** Primer sequences used for PCR real-time analysis.
